# Biosignal PI, an Affordable Open-Source ECG and Respiration Measurement System

**DOI:** 10.3390/s150100093

**Published:** 2014-12-23

**Authors:** Farhad Abtahi, Jonatan Snäll, Benjamin Aslamy, Shirin Abtahi, Fernando Seoane, Kaj Lindecrantz

**Affiliations:** 1 School of Technology and Health, Royal Institute of Technology, Alfred Nobels Allé 10, Stockholm SE-141 52, Sweden; E-Mails: jsnall@kth.se (J.S.); aslamy@kth.se (B.A.); shirin.abtahi@gmail.com (S.A.); fsm@kth.se (F.S.); kaj.lindecrantz@sth.kth.se (K.L.); 2 Academy of Care, Wellbeing and Welfare, University of Borås, Allégatan 1, Borås SE-501 90, Sweden; 3 Department of Clinical Science, Intervention and Technology, Karolinska Institutet, Hälsovägen 7, Stockholm SE-141 57, Sweden

**Keywords:** affordable ECG, Raspberry PI, ADAS1000 analog front-end, open-source, respiration monitoring, thoracic bioimpedance, Medical device development

## Abstract

Bioimedical pilot projects e.g., telemedicine, homecare, animal and human trials usually involve several physiological measurements. Technical development of these projects is time consuming and in particular costly. A versatile but affordable biosignal measurement platform can help to reduce time and risk while keeping the focus on the important goal and making an efficient use of resources. In this work, an affordable and open source platform for development of physiological signals is proposed. As a first step an 8–12 leads electrocardiogram (ECG) and respiration monitoring system is developed. Chips based on iCoupler technology have been used to achieve electrical isolation as required by IEC 60601 for patient safety. The result shows the potential of this platform as a base for prototyping compact, affordable, and medically safe measurement systems. Further work involves both hardware and software development to develop modules. These modules may require development of front-ends for other biosignals or just collect data wirelessly from different devices e.g., blood pressure, weight, bioimpedance spectrum, blood glucose, e.g., through Bluetooth. All design and development documents, files and source codes will be available for non-commercial use through project website, BiosignalPI.org.

## 1. Introduction

An aging population—an increased percentage of elderly individuals in the overall population—is challenging the current healthcare system by increasing costs, creating a lack of healthcare personnel, and contributing to more complex combinations of chronic diseases [1]. In addition, the spreading of a western lifestyle—low physical activity, combined with a high calorie diet, rich in fat and sugar—has become associated with chronic diseases such as diabetes and cardiovascular diseases in industrialized countries [2,3]. This trend is now noticeable even in developing countries and hence demands for healthcare are expected to rise in the near future [4]. Improvement of healthcare and management of chronic diseases with new methods is the aim of many multidisciplinary researches. These researches include pre-clinical animal trials and clinical human trials of new screening, diagnosis, intervention and treatment methods. These projects typically involve physiological and biological measurements e.g., blood pressure, blood glucose, weight, body composition, activity monitoring, and electrical cardiac activity through electrocardiogram (ECG).

Telemedicine and homecare is a special case, utilising recent advances in information technology (IT). It is sometimes considered a potential solution for increasing patients' quality of life by expanding the accessibility of healthcare and even optimal distribution of healthcare costs [4]. However, these ideas are neither fully proven nor discarded. One of the reasons is the diverse set of measurements and IT systems needed for each individual project. Different sensors and analogue front-ends are required for physiological monitoring of each target patient group, thus demanding application specific biomedical measurement and IT systems. Development of such diverse systems makes technical development of studies in homecare/telemedicine costly and, in particular, time consuming. Subsequently, less time and effort is remaining to engage healthcare personal and target patients.

A flexible platform for fast prototyping of systems in these scenarios can be very useful in pilot projects and for proof of concept. Another benefit of such flexible platforms is for education purposes in the field of biomedical engineering, allowing students to get familiarized with the whole chain of acquisition, processing and presentation of biological signals through a hands-on approach early on the educational program. The best example of such an educational platform is Gamma Cardio (openECG) [5], this open licence project along with a textbook [6] can be used by students to explore the process of medical devices developments. There are other open source projects like OpenMind [7], OpenEEG [8] and OpenBCI [9] which can provide enormous learning resources. However, all of these projects have a limited number of channels and in particular are designed for specific biosignals like EEG with minimum flexibility for covering more measurements. In addition, they are not standalone device and to be functional need a PC, laptop or mobile phone for visualization and analysis of signals which make the whole solution more costly.

The intention of this work is to design an open-source, flexible and affordable framework for developing safe biosignal measurement devices. This framework, we call it Biosignal PI, can be used by researchers, students, and engineers, or even hobbyists, without a deep knowledge of embedded systems, measurement technology or biomedical instrumentation. This framework is modular and electrically safe, and it fulfils many medical standards. ECG has been widely applied for diagnosis and monitoring heart disease, in monitoring the autonomic nervous system through heart-rate variability (HRV) and also for various sports-training applications. Therefore an ECG and respiration measurement module developed as a first example for developing and evaluating the design [10]. This prototype is developed further as Biosignal PI project.

## 2. Constrains

Flexibility is a key feature of a biosignal measurement prototyping framework. It should be scalable for different demands in different projects while still providing high reliability. Each biosignal has specific requirements such as sampling rate, frequency range, specific amplification and safety constrains as specified by relevant medical standards.

## 3. System Design

### 3.1. Embedded Platform

**Figure 1.**
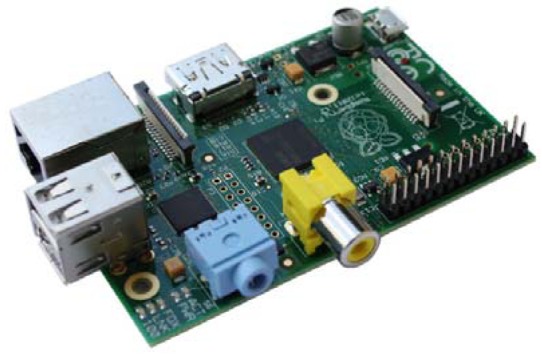
The Raspberry PI Model B (source: Raspberry PI website).

During the last decade, development of microelectronics has provided smaller, faster and more affordable computational platforms. Since flexibility is the main constrain for Biosignal PI, the chosen embedded platform should provide possibility for modular development of hardware and software. Modular hardware development can be done with microcontroller based systems as is done in Arduino projects [11]. However, systems based on operating systems like Linux can provide higher degree of flexibility and hence are more favourable for this kind of development compare to firmware development for microcontrollers. Several compact single-board computers are introduced and have got popular in the last five years e.g. Raspberry PI (RPI) and BeagleBone Black. RPI [12]—a credit-card-size single-board computer with an ARM processor—was chosen for this project, see Figure 1. The RPI is developed by Raspberry Foundation. The main reasons for choosing RPI over its competitors were the affordable price and the active open-source community with enormous amount of projects, shields, and tutorials.

RPI comes in three models; A, B and recently B+. All models use the same CPU and GPU, the difference is in the RAM size and ports. It was initially designed to be an affordable compact computer, supporting students in studies in computer science. However, the presence of a general purpose input/output port (GPIO) made it a popular platform for development of many embedded projects. Model B, the type used in this project, provides an Ethernet port, two USB and one HDMI ports, audio and video outputs; and it has a 700 MHz CPU, GPU, 512 MB RAM and SD card slot. RPI supports several Linux distributions e.g., Raspbian; Debian based distribution optimized for the Raspberry PI hardware. Since it runs on Linux operating system, the programming language is not limited in anyway but Python, C/C++ and Java are among the more popular in the RPI community. Recently, RPI is also supported by Simulink which opens for new ways of learning embedded programming concepts without coding [13]. It is easy to set up a light web server e.g., Lighttpd and Apache, a database server e.g., SQLite, MySQL for specific applications.

### 3.2. Electrocardiogram and Thoracic Bioimpedance Analog Front-End

As mentioned before, an ECG and respiration monitoring system is chosen as the first example in the development of Biosignal PI. Respiration can be recorded via measurement of bioimpedance, *i.e.*, by injecting a small current across the thorax and then sense the corresponding voltage drop. During inhalation, thorax expands, and as air fills the lungs the bioimpedance increases as the conductive surface for the current increases. During exhalation the bioimpedance decreases again [14]. Acquisition of ECG and thoracic bioimpedance can be done through several approaches, from the use of only discrete electronic components to totally integrated analogue front-ends. Integrated front-ends not only reduce the size and power consumption, it also provide a wide range of extra features like lead-off detection and compliance to medical standards such as AAMI EC11, AAMI EC38, IEC 60601-1, IEC 60601-2-25, IEC 60601-2-27 and IEC 60601-2-51. The main competitors for ECG front-ends are the ADAS1000-X from Analog Devices [15] and the ADS129X from Texas Instruments [16]. Both series have almost comparable specifications. ADS1298 can provide eight channels of ECG signals in one chip, good for development of a more compact and slightly cheaper 12-lead ECG device, compared to ADA1000 that has maximum five channels. Nonetheless ADAS1000 (ADAS) was chosen in this work, mainly because ADS1298R is only available in a NFBGA package. For prototyping manual mounting can be crucial and the LQFP package of ADAS is much easier to handle than NFBGA packaging.

**Figure 2.**
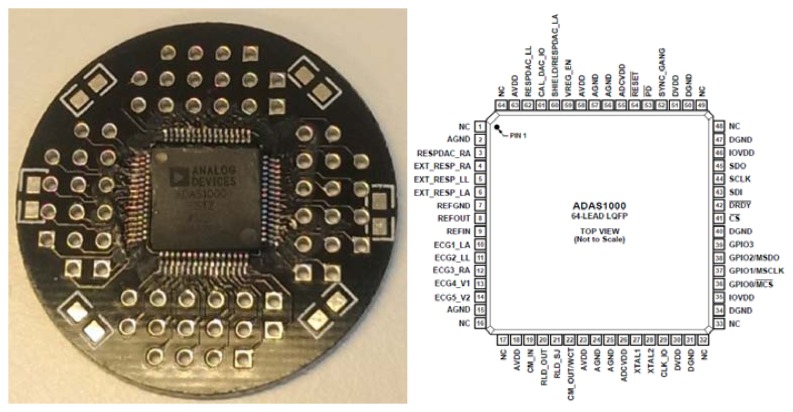
The ADAS1000BSTZ with LQFP64 package is depicted on the right and the one placed on the LQFP adapter (used in a first prototype with in-house manufactured PCB) is on the left.

The ADAS can provide a sample rate of up to 128 kHz and is suitable for portable battery-operated devices, bedside patient monitoring, portable telemetry, and home monitoring systems. ADAS chips can be used in gang mode to provide more ECG channels [15]. In this work, one/two ADAS1000BSTZ is used—that is, the five-channel version that includes all features—as a master and optional slave to provide 8-12 leads ECG in version A and B, respectively. Alternatively, a more affordable version ADAS1000-2BSTZ can be used as slave chip. The chip is used with LQFP 64 pin package, see Figure 2. It is worth to mention that manual soldering of LQFP64 package is relatively difficult and needs some experience and high level of soldering skills.

Typically, a 12-leads ECG uses nine electrodes and right-leg drive (RLD). Three electrodes connected to limbs; right arm (RA), left arm (LA) and left leg (LL) and the remaining six electrodes, named V1-V6, and are positioned on well-defined locations on the chest. Table 1 summarises the composition of the typical 12-leads ECG system. The calculation of aVR, aVL and aVF leads are not done by ADAS, they will have to be calculated as part of the subsequent processing. The V1 and V2 channels can be configured to work as either ECG input or as auxiliary input to perform other measurements.

Respiration measurements are done through thoracic bioimpedance measurements at a programmable frequency from 46.5 kHz to 64 kHz. Respiration measurement can be done on one of the limb leads (Lead I, II or III) or through separate leads connected a pair of dedicated pins [15]. Extended information about principles and applications of bioimpedance measurements is available in [17].

ADAS provides lead-off detection by injecting a DC or an AC current, to monitor the changes in the voltage to detect if an electrode is no longer connected to the patient. The detection has a delay which is below 10 ms for the AC mode, in the DC mode the delay depends on the programmed current and the cable capacitance.

### 3.3. Communication between RPI and ADAS

Communication between analogue front-ends, other integrated circuits, and RPI can be done through different ports e.g., serial peripheral interface (SPI), inter-integrated circuit (I^2^C) and universal asynchronous receiver/transmitter (UART). ADAS uses SPI which requires four links to communicate between one master and several slaves, a clock signal (SCLK) for synchronization, a slave select signal (SSn) and two data lines: master-out-slave-in (MOSI) and master-in-slave-out (MISO). Communication is controlled by the master, which selects the slave, activates the clock, and generates information on MOSI while it samples the MISO [18]. In this prototype, RPI acts as the master, communicating with one/two ADAS as slave.

### 3.4. Defibrillator and ESD Protection

In applications with risk of defibrillation e.g., in intensive or in emergency care protection against overvoltage is required. In other fields of application it is still recommended, as it may protect the device from other types of electrostatic discharge (ESD). The ESD protection layer is designed according to the recommendations in the ADAS datasheet [15]. The protection circuit is based on SP720, which provides protection up to 8 kV against ESD and other transient overvoltage events [19].

### 3.5. Electrical Safety Insulation Layer

Electrical safety is among the most important requirements in medical device design. International Electrotechnical Commission (IEC) standards cover two types of insulation for user protection, IEC 60601 and IEC 60950. To prevent cause electric shock, cardiac arrhythmias, burns, or even damage to internal organs [20], the user (patient/operator) should be isolated from the high-voltage parts of the system, and the leakage currents must be kept low.

**Figure 3.**
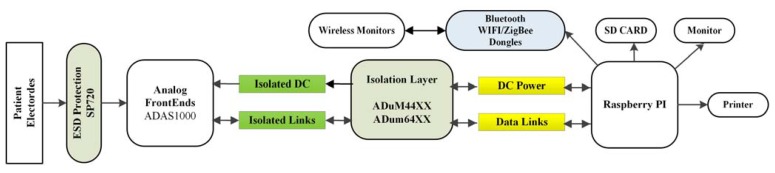
System Architecture: Analog front-end, Raspberry PI (RPI), defibrillation protection, and patient isolation and RPI peripherals. Signals collect and process by RPI, and results can be shown on a monitor or printer or be saved on an SD card; data can even sent/received by Bluetooth, Wi-Fi or ZigBee to/from another device or cloud service.

Isolation can be implemented at different levels. For applications with no connection, direct or indirect, to the power-line voltage e.g., battery-driven Holter devices the problem is automatically solve. However, as there may be a need for connecting RPI to peripherals e.g., printer, monitor, LAN, a proper isolation is included into the design. Isolation is achieved by insulating both the data (SPI) and DC power links between ADAS and RPI, as illustrated in Figure 3.

Optocouplers are typical components use to achieve insulation, signals are transferred between isolated parts and none isolated part using light. An alternative is iCoupler technology, combining high-speed CMOS and monolithic air core transformer technology, which allows lower cost, size, power, and higher reliability compared to optocouplers [21]. The SPI and DC lines are isolated by using the ADuM64XX and ADuM44XX family from Analog Devices. They provide 5 kV isolation, thus comply with IEC 60601 and IEC 60950. The ADuM6200 provides isolated DC power, and ADuM4400 gives isolated digital communication, allowing a bit rate of 90 Mbps [22–24].

### 3.6. Software Development

As discussed before, RPI provides plenty of freedom of choice regarding operating system and programming language. In this work Raspbian Linux and C++ were chosen for implementation of software that initializes the ADAS, retrieves the signals from it, and visualizes and records the signals in a desired format. For the graphical user interface (GUI) development Qt, a cross-platform application framework using standard C++, is used. The Qt also facilitates multi-threading. The Qt is a very popular framework with excellent documentation and useful examples [24]. As compiling an application on the RPI could slow down development process, cross compilation on a PC with Ubuntu OS [18] is used to produce executable code for the RPI platform. In order to achieve the required sampling rates above 2 kHz while the signals are plotted, sampling is done in an independent thread from GUI. Communication between these two threads is done by a method from Qt called signal & slot. This mechanism uses a queued connection which means that the signal is placed in the GUI threads event loop and the GUI is allowed to finish its current task before the slot is invoked [25].

Development of software for medical devices is regulated by several standards, such as ISO 13485, EN ISO 14971 and IEC 62304. These standards cover quality management systems, risk management and software lifecycle processes of medical devices, respectively [26]. Since the software development of this project does not meet any of these standards, it should be considered as software of unknown pedigree (SOUP). Any clinical use should be done after ensuring safe and reliable performance of the device. All responsibilities for this lay on the user, the authors of this paper assume no liabilities for the use of this material.

### 3.7. Biosignal PI Architecture

Figure 3 shows the proposed system architecture for Biosignal PI. The system includes RPI as a computational module, a digital and DC power isolation layer, electrostatic discharge (ESD) protection for body electrodes connected to analogue biosignal front-ends. Different biosignals and vital sign monitoring modules can be added by proper front-ends or as wireless monitors through Bluetooth, WiFi, or ZigBee. RPI may be connected to different peripherals such as monitor, printer and keyboard and even available shields for RPI depend on each demands of each project. RPI and potential peripherals are not designed as medical device and hence insulation layer is used to isolate the breakout board from RPI. Even if insulation and ESD protection characteristics are chosen to comply with requirements for patient safety, no steps towards formal certification have been taken. It is up to anyone who wants to base an MDD or FDA approved device on the Biosignal PI to make sure that all requirements are met.

**Figure 4.**
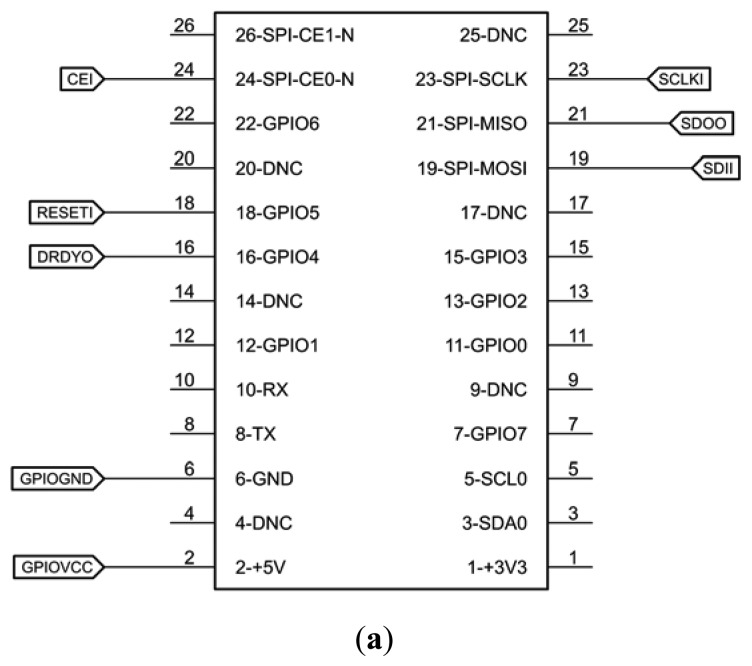

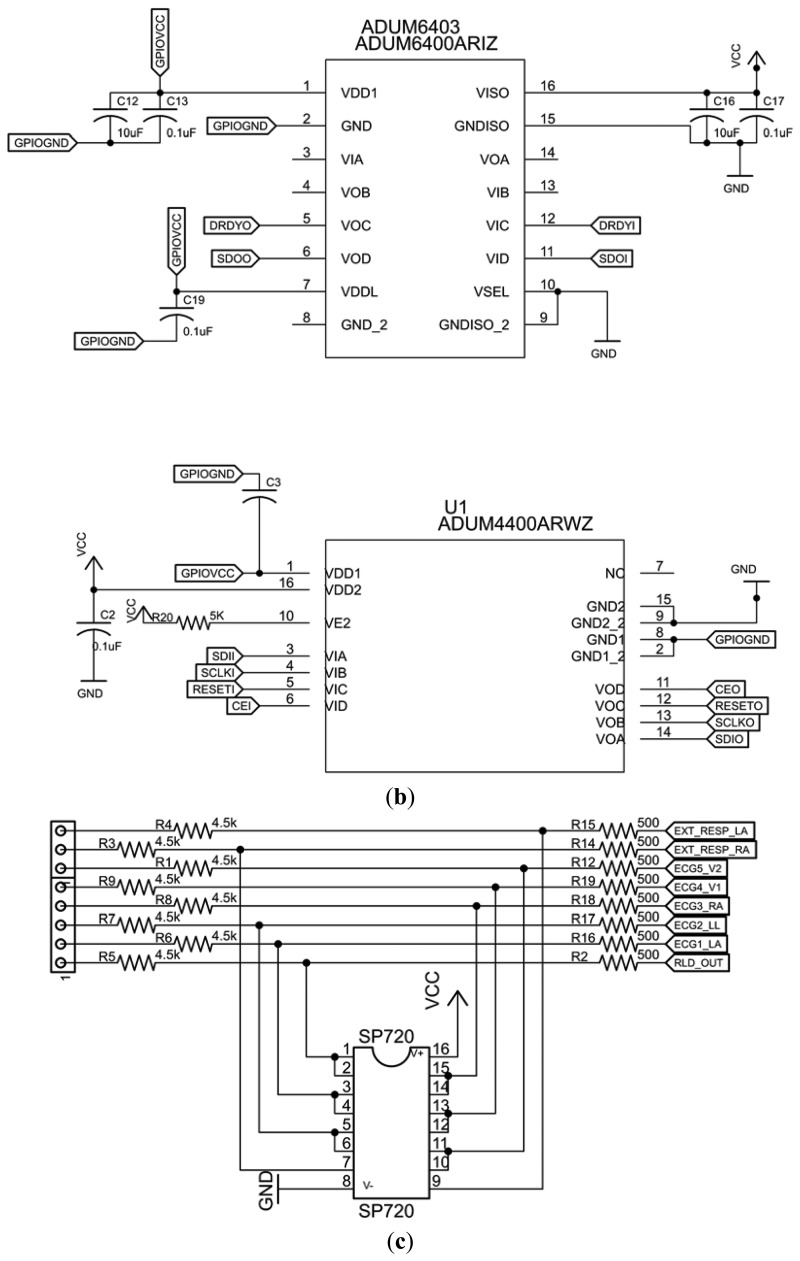

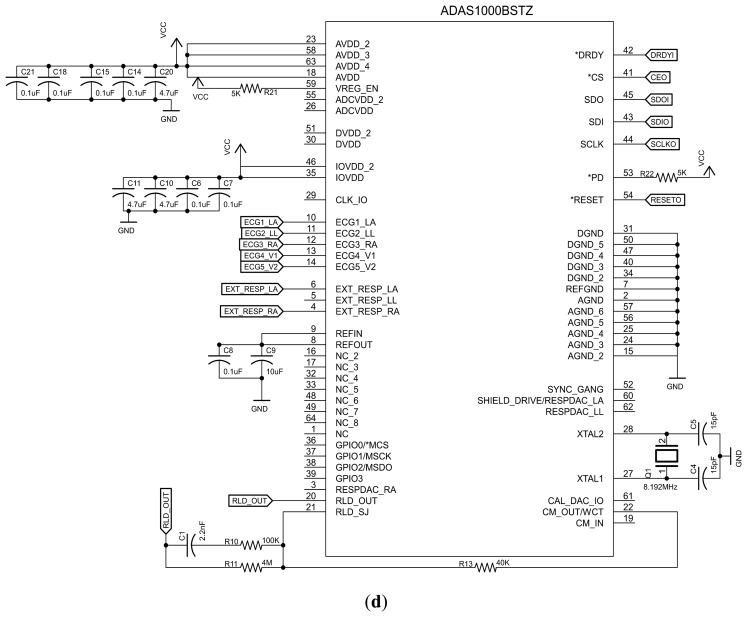
Schematic diagrams of prototype. GPIO Connector of Raspberry PI (**a**), Insulation layer for DC and SPI Communication using ADuM chips (**b**), ESD protection of ECG/external respiratory path by using SP720 (**c**) and ADAS1000 (**d**).

In the first implementation of this architecture, ECG and respiration breakout board was designed for the ADAS1000 chip. The schematic and double-layer printed circuit board (PCB) was designed using the free version of CadSoft Eagle V6.5 [27]. Figure 4 shows the schematic diagram of a 5–8 leads system with all necessary components for operation of ADAS, ESD protection and isolation of board from RPI.

## 4. Results

**Figure 5.**
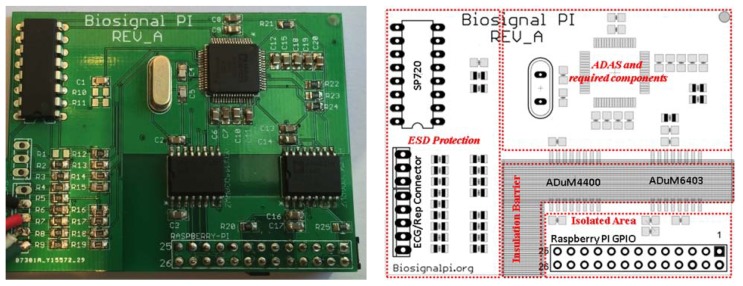
The prototype of Version A; PCB Layout with specified functional blocks (to the right) and assembled PCB (to the left). 4mm air clearance for creepage currents required by IEC 60601 can be recognized by black hachures in the layout and correspondent dark green area (area without copper) in the assembled PCB.

The breakout board was designed in two versions, A and B, providing 5–8 or 12 leads ECG systems, respectively. The final dimensions of the PCB are 60 × 50 × 1.5 mm and 60 × 57 × 1.5 mm for versions A and B, respectively. Version B uses two ADAS chips to provide 12 leads and it also hosts a standard Db15 connector for the ECG cable. The assembled board of version A and its layout is shown in Figure 5; this is a 5–8 leads ECG and respiration version using one ADAS. Table 2 summarizes the main components used in the design and their approximate costs. The total cost exclude RPI is around $60 for the version A and $95 for the version B.

**Figure 6.**
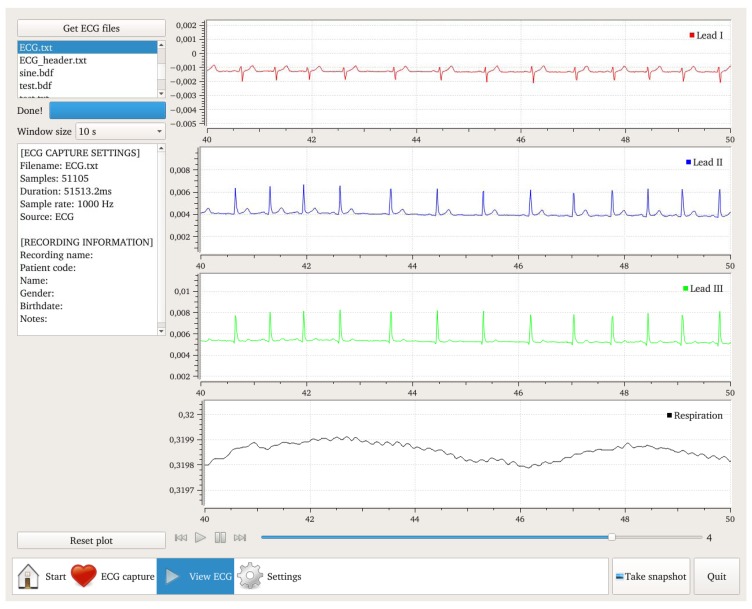
Screenshot of a recorded 3-leads ECG file.

The implemented software can capture ECG and respiration signals at selectable sampling frequencies and visualize them in the real-time. The captured signals can be saved as text file or in European Data Format (EDF) [28]. The software currently provides an interface for browsing the recorded files. A screenshot of the Graphical User Interface (GUI) for browsing the recorded file is depicted in Figure 6. The screenshot illustrates a 10 s epoch of a 3-leads ECG and respiration for a healthy subject. The respiration activity is recorded by using thoracic impedance at 56 kHz through Lead I (right and left arms).

## 5. Discussion

### 5.1. Cost

The total cost of the single prototype breakout board was around $76 for the 5-leads version; higher volumes will yield lower costs. The cost of the final standalone platform *i.e.*, RPI and breakout board is affordable compare to solutions based on PCs and even tablets. In addition, adding other physiological measurements like blood pressure, body weight, and body composition will make the solution more versatile and also economical for homecare and telemedicine. RPI has given us a flexible platform for further development. It is possible to add Bluetooth, Wi-Fi, GSM/3G, printers, cameras, *etc.* simply by using a USB port or GPIO. It is worth mentioning that more USB ports can be added through a USB hub.

### 5.2. Thoracic Impedance Measurements and ADAS1000

The thoracic bioimpedance measurement for monitoring can be done through any of Lead I, Lead II, or Lead III. However, as Lead I measures across both lungs; lead I is the most suitable one for monitoring of respiration. As an alternative, it is possible to use separate electrodes for EBI measurements; these can be placed to optimize respiratory component in impedance signal. ADAS normally uses a two electrode configurations for EBI measurements (using the same pair of electrodes to inject the current and to measure the voltage); this method is sensitive to the skin-electrode impedance of electrodes, and it is not ideal for EBI measurements. Tetra-polar EBI measurement is a robust method that reduces the influence of electrode impedance and skin-electrode contact impedance by using one pair of electrodes for current injection and another pair for voltage measurement. It is possible to configure the ECG independent impedance measurement of ADAS to form a tetra-polar bioimpedance measurement system. This may potentially make ADAS a useful tool for impedance cardiography recording.

### 5.3. Raspberry PI as a Development Platform

In general, RPI can be used as a platform in an affordable homecare setting. Bluetooth, WIFI, GSM/3G/LTE and ZigBee can be used simply by employing USB dongles. Best of all, the RPI is available at a price of less than $60; RPI, including enclosure, power supply, and high-speed SD card. RPI Model B and B+ are fairly compact; however they are not suitable for specific projects like wearable devices.

**Figure 7.**
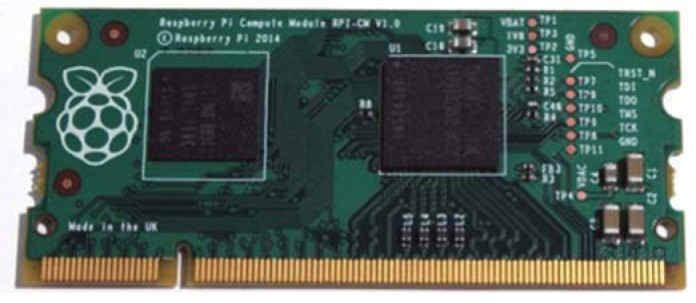
The RPI Compute Model; 67.6 × 30 mm board as DDR2 SODIMM (Source: Raspberry PI website).

A recently introduced version, called Compute Module is aim to overcome this limitations. This module includes an RPi (the BCM2835 processor and 512 MB of RAM), 4 GB eMMC Flash and is integrated on to a small 67.6 × 30 mm board which fits into a standard DDR2 SODIMM connector. RPI Compute Module is depicted in Figure 7.

RPI is now facing a lot of competition, e.g., BeagleBone Black, Goosberry, UDOO, DragonBoard and Hackberry. These competitors usually provide better performance in terms of faster processors, bigger memory size, and more GPIO pins. The developed hardware is compatible with these platforms, and just need an adapter for connecting GPIO pins to correspondent pins of Biosignal PI. Since we have used the Qt development framework, porting the developed software to a new platform could be possible. The most important needed modification is GPIO communication module which is now based on Broadcom BCM2835 and should be modified according to the target platform.

### 5.4. Standard Compliances

As mentioned before, medical device functionality and safety of an ECG device is covered by several standards. The use of ADAS for the design gives free compliance to requirements for diagnostic ECG device. Table 3 summarizes the relevant standards and how they are covered in this design. Some of standards like EN 1041, EN 980 and EN 1041 are more related to manufacturing and support of a commercial device which is not covered by this project yet. This is not meant to be a full description of medical standards; more information can be found on relevant references like [6].

### 5.5. Potential to Develop as a Wearable System

The proposed Biosignal PI system is relatively compact as a standalone device, but there is a trend towards even more compact and wearable devices for physiological measurements and wireless links to present data on mobile phones and smart watches. These products are usually not designed to act as main diagnostic tool by providing healthcare grade applications, they often intended for fitness and sport. For such wearable solutions, which in nature need more compact and different form factor than RPI Model B/B +, RPI Compute Module can be used. Further development can miniaturize the system in a way that it can be a part of personalized wearable health-monitoring solutions. Advances in textile electronics and printed electronics have demonstrated good performances for dry textile-based electrodes and printed electronics [29,30]. The ultimate solution might be a wearable system, such as the sensorized garments in the ATREC project [31] and the Textrode straps for impedance cardiography [32].

### 5.6. Laboratory Work, Design-Building Experiences and Learning Tool

The Raspberry PI foundation has provided an educational platform for kids. In addition, open-source projects and shields are developed by RPI community for home automation, robotics, *etc.* Further development of Biosignal PI can provide an educational platform for engineering students. Biomedical engineering is a multidisciplinary discipline integrating know-how from different fields and is likely that not all the students master embedded systems with enough proficiency to build the embedded system equivalent to RPI from scratch to implement a specific biomedical application. Biosignal PI together with some laboratory exercises could facilitate the implementation of teaching-learning activities focused on the acquisition and analysis of physiological signals [33]. This could be of particular interest in programs implementing the CDIO concept (Conceive-Design-Innovate and Operate) with a significant load of student centred learning specifically through Design-Build experiences [34].

### 5.7. Potential Future Development

The implemented software functions have demonstrated the features of lead-off detection, signal acquisition, storage as text or EDF file, illustration, and signal processing of the platform. Further software development will provide more controls over ADAS's registers and processing options, such as heart-rate variability analysis. Transmission of signals over Bluetooth will also implement and uses as link with Bluetooth enabled devices such as some pulse oximeters, blood pressure devices, and weight scales. To explore educational potential of RPI for education of biomedical engineering, alongside with hardware/software developments; syllables and instructions for laboratory exercises and assignments of such courses is needed.

## 6. Conclusions/Outlook

An ECG and respiration measurement device has been developed as first step of Biosignal PI, a flexible compact platform for biosignal measurements. Further development of this platform will provide a flexible framework for fast prototyping and proof of concept in animal and human clinical studies or as a part of a personalized health-monitoring and homecare systems. It could also be developed as an affordable and versatile educational platform, in a similar manner that Arduino has contributed to facilitate learning of programming and embedded systems in the field of electronics [35–38].

## Figures and Tables

**Table 1.** Leads name and composition of ECG leads by using one or two ADAS [15].

**ADAS**	**Lead Name**	**Composition**
*Master*	I	LA – RA
	II	LL – RA
	III	LL – LA
	aVR	RA – 0.5 × (LA + LL)
	aVL	LA – 0.5 × (LL + RA)
	aVF	LL – 0.5 × (LA + RA)
	V1	V1 – 0.333 × (LA + RA + LL)
	V2	V2 – 0.333 × (LA + RA + LL)

*Slave*	V3	V3 – 0.333 × (LA + RA + LL)
	V4	V4 – 0.333 × (LA + RA + LL)
	V5	V5 – 0.333 × (LA + RA + LL)
	V6	V6 – 0.333 × (LA + RA + LL)

Note: aVR, aVL and aVF are not provided by ADAS and should drive by calculation.

**Table 2.** Components cost.

**Component**	**Cost ($)^a^**	**Cost ($)^b^**
*ADAS1000BSTZ*	35	22.5
*ADAS1000-2BSTZ^c^*	20	13.5
*ADuM4400*	7.5	4
*ADuM6403*	13	8
*SP720, 4kV diode array*	3.1	1.55
*PCB Manufacturing*	5.4	3.45
*Resistors, crystal, capacitors, connectors and etc.*	10	6

Total Cost	94	58

a Cost for low volumes;

b Cost for batch of 1000;

c Optional as slave for 12-leads (Version B).

**Table 3.** Related standards and compliance of current version.

**Standard**	**Topic**	**Covered by**
*AAMI EC 11*	Diagnostic electrocardiographic devices requirements	ADAS

*EC 13*	Minimum safety and performance requirements for electrocardiograph (ECG) heart rate and waveform monitors	ADAS

*EC 38*	Particular requirements for the safety, including essential performance, of ambulatory electrocardiographic systems	ADAS

*EN 1041*	Information from supplier	------

*EN 980*	Device Labelling	------

*IEC 61000*	Immunity to ESD and end electrical burst	SP720
		Creepage air clearance

*IEC/EN 6061-1-1*	General requirements of safety	ADAS
		ADUM

*IEC/EN 6061-1-2*	Electromagnetic compatibility requirements and test	-Ground Planes
		-Component Segregation
		-Decoupling Capacitors
		-Separation of traces

*IEC/EN 6061-2-25*	ECG Safety requirements	ADAS

*IEC/EN 6061-2-27*	Particular requirements for the basic safety and essential performance of electrocardiographic monitoring equipment	ADAS

*IEC/EN 6061-2-51*	Particular requirements for safety, including essential performance, of recording and analysing single channel and multichannel electrocardiographs	ADAS

*ISO/EN 10993-1*	Biological Evaluation	------

*ISO/EN 14971*	Risk Management	------

*ISO 13485*	Quality Management System	------

*ISO 62304*	Software lifecycle processes of medical devices	------
